# P2X7 Receptor Triggers Lysosomal Leakage Through Calcium Mobilization in a Mechanism Dependent on Pannexin-1 Hemichannels

**DOI:** 10.3389/fimmu.2022.752105

**Published:** 2022-02-09

**Authors:** Stephanie Alexia Cristina Silva Santos, Pedro Muanis Persechini, Bianca Monteiro Henriques-Santos, Victória Gabriela Bello-Santos, Newton G. Castro, Júlia Costa de Sousa, Fernando Ariel Genta, Marcelo Felippe Santiago, Robson Coutinho-Silva, Luiz Eduardo Baggio Savio, Eleonora Kurtenbach

**Affiliations:** ^1^ Laboratory of Molecular Biology and Biochemistry of Proteins, Biophysics Institute Carlos Chagas Filho, Federal University of Rio de Janeiro, Rio de Janeiro, Brazil; ^2^ Laboratory of Immuno-Biophysics, Biophysics Institute Carlos Chagas Filho, Federal University of Rio de Janeiro, Rio de Janeiro, Brazil; ^3^ Laboratory of Insect Physiology and Biochemistry, Oswaldo Cruz Institute – Oswaldo Cruz Foundation (IOC-FIOCRUZ), Rio de Janeiro, Brazil; ^4^ Laboratory of Molecular Pharmacology, Institute of Biomedical Sciences, Federal University of Rio de Janeiro, Rio de Janeiro, Brazil; ^5^ Laboratory of Immunophysiology, Biophysics Institute Carlos Chagas Filho, Federal University of Rio de Janeiro, Rio de Janeiro, Brazil

**Keywords:** lysosomes, P2 receptor, cathepsins, extracellular ATP, lysosomal permeabilization

## Abstract

The P2X7 receptor is a critical purinergic receptor in immune cells. Its activation was associated with cathepsin release into macrophage cytosol, suggesting its involvement in lysosomal membrane permeabilization (LMP) and leakage. Nevertheless, the mechanisms by which P2X7 receptor activation induces LMP and leakage are unclear. This study investigated cellular mechanisms associated with endosomal and lysosomal leakage triggered by P2X7 receptor activation. We found that ATP at 500 μM and 5 mM (but not 50 μM) induced LMP in non-stimulated peritoneal macrophages. This effect was not observed in P2X7-deficient or A740003-pretreated macrophages. We found that the P2X7 receptor and pannexin-1 channels mediate calcium influx that might be important for activating specific ion channels (TRPM2 and two-pore channels) on the membranes of late endosomes and lysosomes leading to LMP leakage and consequent cathepsin release. These findings suggest the critical role of the P2X7 receptor in inflammatory and infectious diseases *via* lysosomal dysfunction.

## Introduction

Endosomes are membrane-surrounded organelles that transport extracellular macromolecules in eukaryotic cells (1). Plasma cell membrane invaginations originate primary endocytic vesicles that deliver their contents to early endosomes in the cytoplasm. Early endosomes mature and form late endosomes. The late endosomes can fuse with lysosomes to form a transient hybrid organelle called the endolysosome (1). Larger particles such as pathogens and apoptotic cells are usually internalized by phagocytosis—a receptor-mediated cell mechanism of engulfment and uptake of particles within a plasma membrane-derived vesicle. These vesicles called phagosomes can also fuse with lysosomes forming phagolysosomes (2).

The maturation of endosomes/phagosomes and their fusion with lysosomes is an essential homeostatic process that allows the degradation of endocytic content (e.g., macromolecules or pathogens) by over 50 lysosomal hydrolases (3). These processes are regulated by ion channels present in the lysosomal membrane, including the TRP family channels (TRPMLs, TRPM2, TRPA1), the two-pore channel (TPC) family channels (TPC1, 2), and possibly P2X purinergic receptors (4–6). These ion channels are essential for maintaining the acidic pH in the lumen of these organelles, supporting the activity of proteases such as cathepsins and the degradation of engulfed/captured cargo (3, 6). However, when these ion channels in lysosomes do not function correctly, the organelles become dysfunctional. This phenomenon is associated with pathological conditions such as neurodegenerative, autoimmune, and infectious diseases (7).

Endolysosomal/phagolysosomal membrane permeabilization or leakage promotes the release of organelle content into the cytosol, increasing cytosolic acidity and triggering cell signaling mechanisms of inflammation and cell death. The discharge into the cytosol of pathogen-associated molecular patterns and host proteases (e.g., cathepsin B and cathepsin L) induces the activation of the inflammasome and caspases, possibly resulting in apoptotic, pyroptotic, and necrotic cell death through various cellular pathways (8). Despite its relevance in infectious and inflammatory diseases (7), the mechanisms underlying lysosomal permeabilization and leakage remain poorly understood.

The ATP-gated P2X7 receptor is an ion channel involved in several cellular responses in immune and non-immune cells (9–11). Once activated by extracellular ATP, this receptor leads to Ca^2+^ and Na^+^ influx and K^+^ efflux (9, 12). P2X7 receptor-mediated calcium influx and K^+^ efflux are relevant for critical cellular processes, including phagocytosis, apoptosis, inflammasome activation, and cytokine secretion (9, 10, 13–15). This receptor can also stimulate the opening of non-selective pores permeable to organic molecules up to 900 Da depending on the time and ATP concentration of stimulation (12). Furthermore, the P2X7 receptor activation induces the opening of the pannexin-1 and connexin hemichannels to increase the calcium influx and propagate intercellular calcium waves (16). The opening of these hemichannels potentiates the release of ATP into the extracellular medium, amplifying purinergic signaling in a positive feedback loop (17, 18).

Curiously, P2X7 receptor activation was also associated with impairment of lysosomal functions and cathepsin release from macrophages, suggesting the possible involvement of this receptor in lysosomal leakage in phagocytes (19–22). Despite this evidence, the precise mechanisms underlying the possible role of the P2X7 receptor on endolysosomal leakage remain unclear (22). Therefore, in this study, we investigated cellular mechanisms associated with endosomal and lysosomal leakage triggered by P2X7 receptor activation. Our unique results suggest that the P2X7 receptor- and pannexin-1 hemichannel-mediated calcium influx is essential for lysosomal leakage/permeabilization and consequent cathepsin release possibly by activating specific ion channels (TRPM2 and TPC channels) on the membrane of these organelles. These findings are compelling because changes in lysosomal homeostasis are associated with inflammatory and infectious diseases such as SARS-CoV2 infection and killing of intracellular mycobacteria (7, 23, 24).

## Material and Methods

### Animals and Reagents

We used C57Bl/6 Wild-type mice (P2X7^+/+^, WT) and P2X7 knockout mice (P2X7^-/-^ from Jackson Laboratories – JAX stock #005576 Mice and Services, Bar Harbor, ME, USA). P2X7 knockout mice that are homozygous for the targeted allele (P2X7R ^Δ506–532^) are viable, fertile, normal in size, and do not display any gross physical or behavioral abnormalities (25). The procedures for the care and use of animals were according to the Brazilian College of Animal Experimentation guidelines. The Commission for the Ethical Use of Research Animals from the Federal University of Rio de Janeiro (UFRJ) approved all experiments (protocol number: IBCCF166/18). Roswell Park Memorial Institute Medium 1640 (RPMI), Lucifer Yellow (LY), Lysotracker Green (LG), mefloquine (MFQ), adenosine-5’-triphosphate disodium salt hydrate (ATP), ethylene glycol-bis(2-aminoethylether)-N,N,N′,N′-tetraacetic acid (EGTA) and P2X7 antagonist A740003 were obtained from Sigma-Aldrich (St. Louis, USA). Fetal bovine serum (FBS) and penicillin-streptomycin were purchased from Gibco-BRL (São Paulo, SP, Brazil). BAPTA-AM and probenecid were from Invitrogen Molecular Probes (Eugene, OR, USA). Magic Red Cathepsin B Assay was from ImmunoChemistry (Bloomington, USA). Phosphate buffer solution (PBS) (137 mM NaCl, 2.7 mM KCl, 10 mM Na_2_HPO_4_, 2 mM KH_2_PO_4,_ pH = 7.4), Z-Phe-Arg-AMC (Z-FR-AMC) (C9521), and Z-Arg-Arg-AMC (Z-RR-AMC) (C5429) were from Sigma-Aldrich (St. Louis, USA). Hoechst 33342 was from Life Technologies (Eugene, OR. USA). Goat anti-mouse pannexin-1 antibody (K-20) (Santa Cruz Biotechnology, CA, USA), anti-vinculin monoclonal antibody (Sigma Aldrich, Missouri, USA), mouse anti-goat IgG (H+L)-Alexa Fluor^®^ 546 and mouse anti-goat IgG-HRP secondary antibodies (ThermoFisher Scientific, MA, USA), fura-2 AM (Thermo Fisher Scientific, MA, USA), ECL Plus Western Blotting Detection System (GE Healthcare Life Sciences, NY, USA), SuperSignal™ ELISA Femto Substrate (Thermo Fisher Scientific, MA, USA).

### Preparation of Peritoneal Macrophages

Non-stimulated peritoneal macrophages were obtained from C57Bl/6 WT or P2X7^-/-^ male mice, 8–10 weeks old, after peritoneal lavage. Cells were centrifuged at 300 x *g* for 10 minutes and plated at 2 x 10^5^ cells/mL in RPMI-1640 medium for 1 h at 37°C and 5% CO_2_. Then these cells were washed three times with PBS pH 7.4 to remove non-adherent cells, and adherent cells were cultured in RPMI-1640 medium supplemented with 10% FBS and 1% penicillin-streptomycin. The peritoneal macrophages were maintained in culture at 37°C and 5% CO_2_ in culture plates with a diameter of 35 mm and a glass slide of 10 mm in diameter at the bottom divided into four compartments for microscopic observation (Greiner Bio-One, North Carolina, USA). For the enzymatic activity assays, the macrophages were plated on 12-well cell culture plates (ThermoFisher Scientific, MA, USA). Phenotypic analyses were performed by staining the cells with CD11b and F480 macrophage markers, indicating a purity of more than 90%.

### Macrophage Endocytosis Assay

For the endocytosis assay, the peritoneal macrophages were incubated for 45 minutes in RPMI-1640 medium with L-glutamine, without fetal bovine serum at 37°C, 5% CO_2_ in the presence of the following fluorescent probes in independent experiments: 1 mM Lucifer Yellow (495/519) nm, 50 nM Lysotracker Green (504/511) nm or 10 µM Yo-Pro 1 (491/509 nm) (26, 27). In the assays using inhibitors, cells were pretreated (30 minutes) with the following: 100 nM A7400043, 100 µM mefloquine, 50 µM carbenoxolone, 20 µM 10Panx, 150 µM probenecid, and 50 µM 2-APB or 50 µM verapamil. After the endocytosis assay, the cells were washed three times with physiological extracellular solution (145 mM NaCl, 5 mM KCl, 1 mM MgCl_2_, 10 mM Hepes, 1 mM CaCl_2_, pH 7.4) to remove the probe that was not endocytosed. Finally, the peritoneal macrophages were stained with the nuclear dye Hoechst 33342 (350/461 nm) (Life Technologies, OR, USA) or with CellMask™ Deep Red Plasma Membrane Stain (659/676 nm) (Life Technologies, OR, USA). Live cells were kept in physiological extracellular solution (145 mM NaCl, 5 mM KCl, 1 mM MgCl_2_, 10 mM Hepes, 1 mM CaCl_2_, pH 7.4) and observed before and after ATP treatment at various concentrations (50 µM, 500 µM, and 5 mM) or UTP (100 µM) for 10 minutes at 37°C. For the experiments with calcium chelators 2 mM de EGTA e 10 µM de BAPTA-AM were added to physiological extracellular solution. The cells were observed in a confocal microscope - rotating disk Zeiss Cell Observer Yokogawa (Cell Observer SD, Carl Zeiss, located on the Gustavo De Oliveira Castro light optical microscopy platform, from the Carlos Chagas Filho Biophysics Institute - UFRJ). The images were obtained in three dimensions (Z-stack), and in all tests, 14 cellular fields were analyzed per well at 100x magnification.

### Image Processing

The images of live cells were processed using Zen Lite Blue software (Zen Digital Imaging for Light Microscopy), and the Open-Source software ImageJ focused on biological-image analysis (Fiji software). Initially, images were processed to reduce background noise, and measurements of mean fluorescence intensity from a region of interest were performed to assess the fluorescence level only at that point. Fluorescent puncta counts (fluorescent-labelled vesicles) were performed on all sections of the Z-stack image using the Fiji plugin ‘cell counter’. For image composition, orthogonal projections were made, consisting of the union of all sections obtained from the 3D images created for the living cells.

### Cathepsin Enzymatic Assay

Macrophage supernatants were collected before and after each treatment. For the negative control, cells were pre-incubated for 10 min with 100 µM cysteine proteases inhibitor E-64 before adding extracellular ATP. Cells were incubated with cathepsin assay buffer and the various fluorogenic cathepsin substrates [50 µM Z-Phe-Arg-AMC (Z-FR-AMC) (Sigma C9521), 50 µM Z-Arg-Arg-AMC (Z-RR -AMC)] (Sigma C5429). The reading of enzymatic kinetics was performed for 90 min at 37°C excitation/emission of 380/460 nm on a SpectraMax Gemini XPS (Molecular Devices, LLC. First Street San Jose, CA, USA).

### Magic Red™ Cathepsin Assay

The Magic Red™-MR-RR2 kit (Immunochemistry Technologies, USA) was used to quantify and monitor cathepsin B activity in cultured peritoneal macrophages. Magic Red 1:10 has been added directly into the culture (RPMI - not supplemented) containing 2 x 10^5^ cells/mL. When cathepsin is active, this substrate (MR-RR2) is cleaved and the cresyl violet fluorophore becomes fluorescent by excitation 592/628 nm (28). Cells were also incubated with 50 nM Lysotracker green for 30 minutes at 37°C and then incubated for 10 minutes with Hoechst 33342 to detect nuclear morphology. Subsequently, live cells were observed by spinning disk confocal microscopy before and after 5 mM ATP treatment.

### Calcium Imaging

Peritoneal macrophages plated in 25 mm round coverslips were loaded with loading solution containing 4 µM fura-2 AM (Thermo Fisher Scientific, MA, USA), Pluronic 0,04% at 37°C for 45 minutes. The coverslips were mounted in a 200 µl open imaging chamber in a PDMI-2 perfusion incubator (Warner Instruments) maintained at 37°C. Solutions were fed to the incubator with a peristaltic pump: physiological extracellular solution (145 mM NaCl, 5 mM KCl, 1 mM MgCl_2_, 10 mM HEPES, 1 mM CaCl_2_, pH 7.4; control solution); physiological extracellular solution + 5 mM ATP in the absence or presence of 1 mM CaCl_2_ and physiological extracellular solution + 2 mM EGTA, (0-Ca^2+^). Ratiometric imaging was performed through a 20×, 0.45 n.a. objective (Nikon Ti-U) and fura-2 filter set (Semrock) with an Evolve 512 EMCCD camera (Photometrics) and Lambda DG-4 illuminator (Sutter), under control of MetaFluor software (Molecular Devices). Fluorescence image pairs (F340 and F384) were obtained at 0.5 Hz and analyzed with Fiji-ImageJ. The image sequences were corrected for small movement artefacts (field translation), elliptical regions of interest (ROI) were drawn on each cell in the field, and the F340/F384 average intensity ratios (R) within each ROI was calculated without background subtraction. The R traces were further analyzed with Clampfit software (Molecular Devices). The peak changes of intracellular Ca^2+^ (Δ*R*) were estimated as the difference between the maximum *R* value (three-point moving average) during the ATP pulse and the 30-second average value preceding the pulse. For the ATP pulse in Ca^2+^-free medium, during which *R* was still slowly decaying due to recovery from the first ATP pulse, a decaying linear trend was subtracted from each trace before measuring the response. The decay rates were estimated by linear regression in 200-s segments of the *R* traces.

### Immunocytochemistry

After pharmacological treatments described above, samples (2 x 10 ^5^cells/mL) were fixed with 4% paraformaldehyde and 4% sucrose for 15 min at room temperature and blocked with 10% horse serum (Thermo Fisher Scientific, MA, USA and 1% BSA in PBS for 30 min at room temperature. Samples were then incubated (overnight at 4°C) with the following primary antibodies (in 0.1% BSA in PBS): goat pannexin-1 antibody (K-20) (Santa Cruz Biotechnology, CA, USA) diluted 1:500. Cells were then washed and incubated at room temperature for 1 h with the following secondary antibody (diluted 1:300, in 0.1% BSA in PBS): anti-goat IgG (H+L)-Alexa Fluor^®^ 546 (Thermo Fisher Scientific, MA, USA). Finally, samples were mounted with Prolong Gold Antifade Mountant with DAPI (Invitrogen) and examined in a fluorescence microscope Zeiss AxioVert 200M and the three-dimensional images (z-stack) in a Spinning Disk Confocal Microscope ZEISS Cell Observer SD (Peabody, MA, USA).

### Western Blotting

Heart fragments were macerated in HEPES buffer (50 mM Hepes, 10 mM EDTA, 1 mM MgCl_2_, 1% Triton X-100 and protease/phosphatase inhibitor cocktail - pH 7.4) using the Ultra-Turax T25 basic homogenizer (IKA, Germany) at 9500 L/min. This macerate was incubated on ice for 30 minutes and centrifuged at 12000 g for 15 minutes at 4°C. Non-stimulated peritoneal macrophages and SAOS-2 cells were scrubbed in 200 µl of RIPA buffer (20 mM HEPES pH 7.9, 20% glycerol, 200 mM KCI, 0.5 mM EDTA, 0.5% NP-40, 0.5 mM DTT, 1% protease/phosphatase inhibitor cocktail) using a cell scraper, then transferred to a dounce homogenizer. The samples were homogenized on ice for 15 min and left incubating for another 15 min, and then the cell lysate was centrifuged at 15000 g for 10 minutes at 4°C. The supernatant was used for protein dosage by the Bradford method, and 35 μg of protein were resolved in 12% SDS-PAGE at 150 V and transferred to a nitrocellulose membrane (Bio-Rad, CA, USA) for 65 minutes at 350 mA. After transfer, the membrane was blocked with 5% nonfat dry milk in 1X TBS-T [10 mM Tris, pH 7,4, 150 mM NaCl, 0.1% Tween20] for 2 hours and incubated overnight at 4°C with primary antibodies: goat anti-mouse pannexin-1 (K-20) (Santa Cruz Biotechnology, CA, USA) and anti-vinculin monoclonal antibody (Sigma Aldrich, Missouri, USA). The next day the membrane was washed with TBS-T and incubated mouse anti-goat IgG-HRP secondary antibody (ThermoFisher Scientific, MA, USA) for 1 hour at room temperature. After incubation, the washing procedure was repeated and the membrane developed using the chemiluminescent ECL Plus Western Blotting Detection System (GE Healthcare Life Sciences, NY, USA) or SuperSignal™ ELISA Femto Substrate (Thermo Fisher Scientific, MA, USA).

### Statistical Analysis

The data were analyzed using GraphPad Prism version 8.2, and values represent the mean value with standard deviation. Differences between means were assessed using the Student’s t-test, one-way analysis of variance followed or not by the Bonferroni test, all considering p < 0.05.

## Results

### Extracellular ATP Mediates the Leakage of Endosomes and Lysosomes in Macrophages

**Figure 1:**
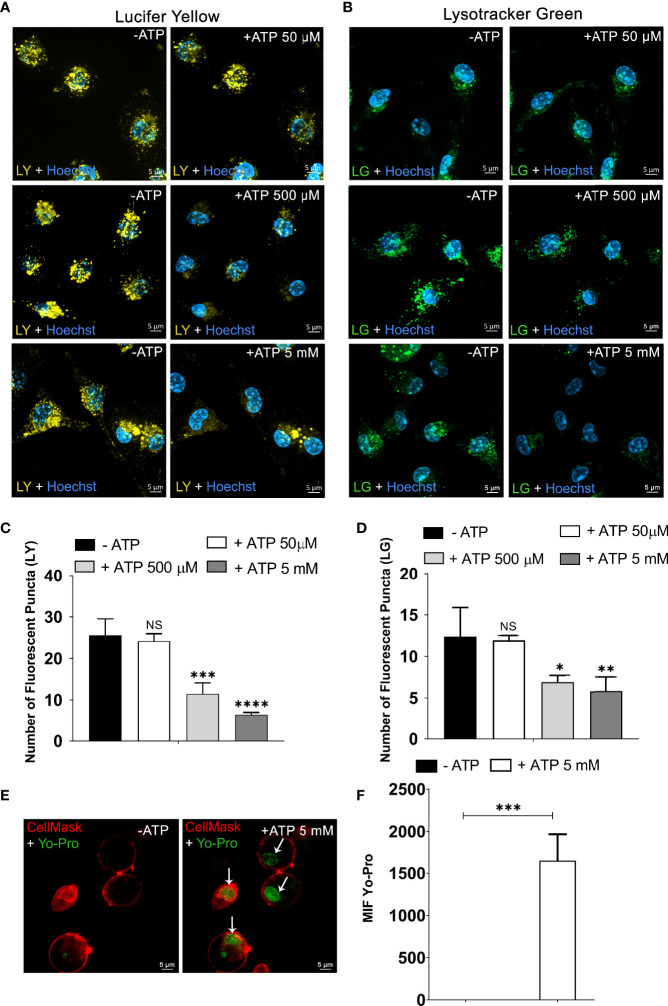
Extracellular ATP Triggers the Rupture of Endosomes/Endolysosomes and Lysosomes. Representative confocal microscope images of peritoneal macrophages from C57Bl/6 WT labeled with **(A)** 1 mM of Lucifer Yellow (LY) and **(B)** 50 nM of Lysotracker Green (LG) for 45 min and then treated or not with ATP (50 µM, 500 µM, or 5 mM) at 37°C for 10 min. **(C)** The number of fluorescent puncta per cell stained with Lucifer Yellow and **(D)** the number of fluorescent puncta per cell marked with Lysotracker Green counted before and after ATP treatment. **(E)** Representative images of peritoneal macrophages labeled with DNA stain YO-PRO^®^-1 for 45 min at 37°C. **(F)** MFI of nuclei stained with YO-PRO^®^-1 from image E was quantified using Zen Lite Blue software with the background subtracted. Cells were visualized using confocal microscopy before and after stimulation with 5 mM ATP at 37°C for 10 min and nuclei stained with Hoechst. Scale bar, 5 µM; original magnification 100x. Data are expressed as mean ± SEM of three independent experiments performed in triplicate. **(C, D)** One-way analysis of variance to compare the number of fluorescent puncta after treatment with ATP vs. the number without treatment. **(F)** T-test analysis to compare the condition of 10 min after treatment with ATP vs. without treatment with ATP. *P < 0.05, **P < 0.01, ***P < 0.001, and ****P < 0.0001. NS, not significant.

First, we investigated the effect of extracellular ATP (50 μM, 500 μM, and 5 mM) on non-stimulated peritoneal macrophage late endosome and endolysosome stability. These intracellular organelles were formed after endocytosis of 1 mM Lucifer Yellow (LY) fluorescent dye (26, 27). Spinning Disk Confocal Microscopy was used to observe continuously the same cell areas before and after eATP treatment. As depicted in **Figure 1A**, 50 μM ATP (concentration that does not activate the P2X7 receptor) did not induce late endosome or endolysosome stability changes. By contrast, 500 μM (*p* < 0.001) and 5 mM ATP (*p* < 0.0001) treatments significantly decreased the number of late endosomes/endolysosomes marked with LY (**Figure 1A**, representative images; **Figure 1C**, quantification), suggesting that P2X7 receptor activation mediates lysosomal leakage. In parallel, a time dependent increase in the fluorescent content of macrophages’ cytoplasm and cell supernatant after the addition of eATP was observed (data not shown). This phenomenon was not detected in macrophages from P2X7 knockout mice. To verify the participation of P2 receptors such as P2Y_2_, macrophages labeled with LY were treated with 100 μM UTP. However, this nucleotide did not diminish the number of LY-stained endolysosomes (**Supplementary Figures 1A, B**).

Next, the fluorescent probe Lysotracker Green was used in endocytosis assays to investigate the ATP effects on lysosomal stability. We observed a significant decrease in the number of lysosomes in peritoneal macrophages treated with 500 μM (*p* < 0.05) and 5 mM ATP (*p* < 0.01) but not with 50 μM ATP (**Figures 1B, C**, representative images, **Figure 1D**, quantification).

To confirm that ATP-induced lysosomal destabilization leads to the release of organelle content to the macrophage cytoplasm, we performed an endocytosis assay with the YO-PRO^®^-1 DNA-staining dye impermeable to the cellular membrane (29). After being exposed to YO-PRO^®^-1 marker for 45 min, non-stimulated macrophage cells were washed three times with PBS to discard the free dye. In the absence of ATP (**Figure 1E**- left), no fluorescence signal related to the YO-PRO^®^-1 dye was detected. However, treatment with 5 mM ATP for 10 min induced YO-PRO^®^-1 escape from endocytic vesicles staining the nucleus (*p* < 0.001) (**Figure 1E**-right). The mean fluorescence intensity quantification of the cell nucleus confirmed that the fluorescence signal was significantly greater after 5 mM ATP treatment when compared in the absence of ATP (**Figure 1F**). The Cell Mask Deep Red Plasma Membrane Stain shows preserved cell membrane integrity.

### P2X7 Receptor Activation Triggers Lysosomal Leakage in Macrophages

The role of the P2X7 receptor in lysosomal leakage was confirmed by performing endocytosis assays with macrophages obtained from P2X7 knockout mice (P2X7^-/-^) and macrophages from wild-type (WT) mice in the presence of 100 nM A740003, a specific P2X7 antagonist.

**Figure 2:**
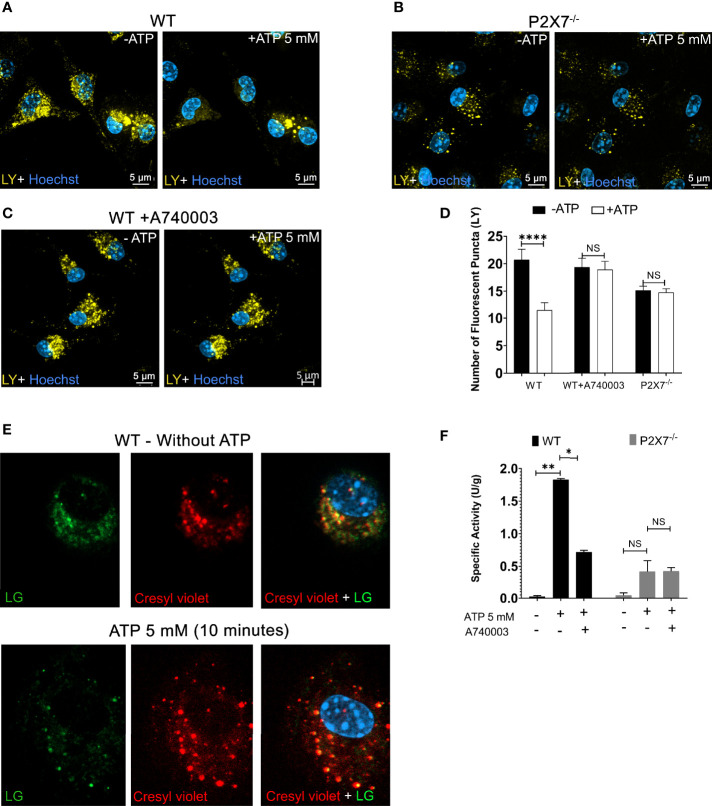
P2X7 Receptor Activation Triggers Endolysosomal Rupture. **(A)** Representative images of peritoneal macrophages of WT preincubated with 1 mM of Lucifer Yellow (LY) for 45 min, **(B)** Peritoneal macrophages from P2X7^-/-^ mice; before and after treatment with 5 mM ATP for 10 minutes, and **(C)** WT macrophages pre-incubated with 100 nM A740003 for 30 minutes. **(D)** The number of fluorescent puncta stained with Lucifer Yellow was counted before and after ATP treatment. **(E)** Representative images of peritoneal macrophages from WT mice incubated with the fluorogenic substrate MR-RR2 given as product cresyl violet fluorophore (red) for cathepsin B activity detection and Lysotracker Green (green) in the absence or presence of 5 mM ATP. **(F)** Cathepsin activity in the supernatants of cells pretreated with 100 nM A740003 and then treated with 5 mM ATP for 10 min. Scale bar, 5 µM; original magnification 100x. Data are expressed as mean ± SEM of three independent experiments performed in triplicates. One-way analysis of variance *P < 0.05, **P < 0.001 and ****P < 0.0001. Analysis of conditions without ATP vs. after treatment with 5 mM ATP. NS, not significant.

Peritoneal macrophages from P2X7^-/-^ animals treated with 5 mM ATP did not show a significant decrease in the number of endolysosomes stained with LY, suggesting an absence of leakage (**Figure 2B**, representative images; **Figure 2D** quantification) when compared to macrophages from WT animals (*p* < 0.0001) (**Figures 2A, D**). The same result was obtained in WT macrophages pre-incubated with 100 nM A740003 for 10 min before 5 mM ATP treatment (**Figures 2C, D**). These results suggest that the effect triggered by 5 mM extracellular ATP is mediated by P2X7 receptor activation.

To determine the effect of P2X7 receptor activation on cathepsin B release from endolysosomes lumen to the macrophage cytoplasm, we used a fluorogenic substrate for cathepsin B (Magic Red MR-RR2). This substrate allows the detection of cathepsin B location and activation. Magic Red MR-RR2 substrate cleavage by cathepsin B in living cells releases the red fluorescent compound cresyl violet as a product (28). Macrophages from WT mice were also previously incubated with Lysotracker Green, allowing the visualization of the colocalization of endolysosomes and cathepsin B product (yellow dots resulting from the merge of the green and red labeling) before 5 mM ATP treatment (**Figure 2E** - left). After ATP treatment, there was a significant decrease in the yellow signal inside lysosomes and a presence of an intense fluorescent red signal dispersed in the cell cytoplasm emitted by the MR-RR2 cleavage product, suggesting the extravasation of cathepsin from lysosomes to the cytoplasm (**Figure 2E** - right).

Cathepsin B enzyme activity assays were also performed on cell supernatants before and after treatment with 5 mM ATP for 10 minutes. Extracellular ATP triggers the exit of active cathepsin B from WT macrophages (**Figure 2F**). This effect was significantly inhibited (*p* < 0.05) in WT macrophages treated with P2X7 receptor antagonist (100 nM A740003) or in macrophages from P2X7^-/-^ mice (*p* < 0.0001) (**Figure 2F**). These results reinforce the notion that P2X7 receptor activation leads to endolysosomal leakage, releasing cathepsin B and endocytosed fluorescent compounds from the lumen of these organelles.

### Pannexin-1 Contributes to P2X7-Mediated Lysosomal Destabilization

**Figure 3:**
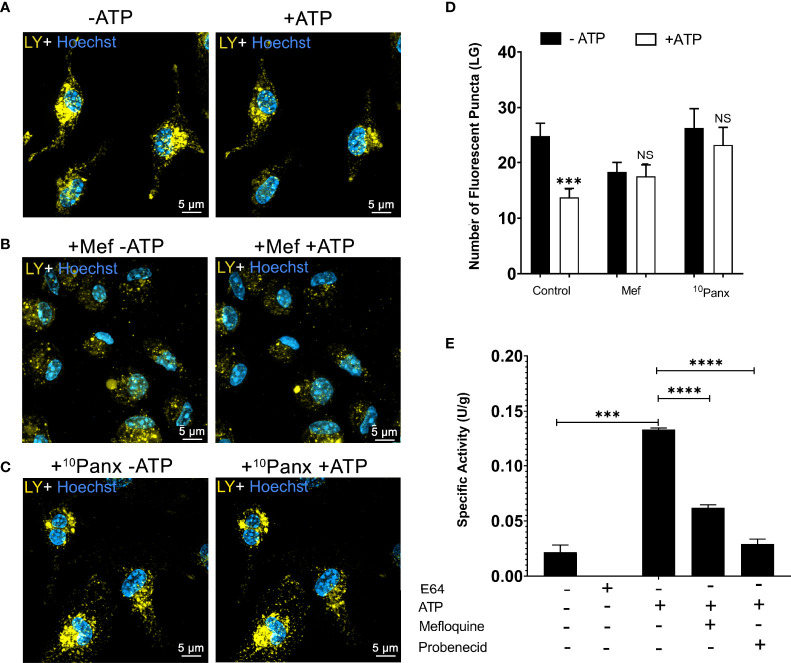
Pannexin-1 Contributes to P2X7-Induced Lysosome/Endolysosome Rupture. Representative images of WT peritoneal macrophages labeled with 1 mM of Lucifer Yellow (LY) for 45 min, **(A)** without treatment with inhibitors, **(B)** WT macrophages pre-incubated with 100 µM mefloquine for 30 minutes, and **(C)** WT macrophages incubated with 20 µM ^10^Panx for 30 minutes; before and after treatment with 5 mM ATP. **(D)** The number of fluorescent puncta stained with Lucifer Yellow was counted before and after ATP treatment. **(E)** Cathepsin activity in the supernatants of cells pretreated with mefloquine, probenecid, and then treated or not with 5 mM ATP for 10 min. Scale bar, 5 µM; original magnification 100x. Data are expressed as mean ± SEM of three independent experiments performed in triplicates and analyzed using one-way analysis of variance ***P < 0.001 and ****P < 0.0001. Analysis of conditions without ATP vs treatment with 5 mM ATP **(D)**. Analysis of conditions without ATP vs treatment with 5 mM ATP and treatment with 5 mM ATP vs mefloquine and probenecid **(E)**. NS, not significant.

Next, we investigated whether pannexin-1 pore participates in lysosomal rupture triggered by P2X7 receptor activation using the pannexin-1 inhibitors mefloquine and 10Panx. We found that peritoneal macrophages pretreated with 100 µM mefloquine (**Figure 3B**) or with 20 µM of the mimetic peptide pannexin-1 ^10^Panx (**Figure 3C**) did not show evidence of lysosomal rupture/destabilization after ATP treatment, as opposed to the control (no ATP) (**Figure 3A**). There was no significant decrease in the number of lysosomes after ATP treatment when cells were pretreated with pannexin-1 pore blockers ^10^Panx or mefloquine (*p* < 0.001) (**Figure 3D**). Pannexin-1 expression in non-stimulated peritoneal macrophages was confirmed by western blot and immunofluorescence analysis (**Supplementary Figures 2A, B**). Cardiac tissue lysates and osteoblasts were used as positive and negative controls, respectively.

Cathepsin B enzymatic assays were also performed in WT macrophages pretreated with 100 µM mefloquine (*p* < 0.001) and 150 µM probenecid as pannexin-1 blockers before ATP treatment (**Figure 3E**). Pannexin-1 inhibition diminished the effects of extracellular ATP on cathepsin B release. These results suggest that pannexin-1 participates in the lysosomal leakage and cell permeabilization triggered by P2X7 receptor activation.

### Lysosome Leakage Depends on Calcium Mobilization *via* P2X7 Receptor and Pannexin-1 Channels

**Figure 4:**
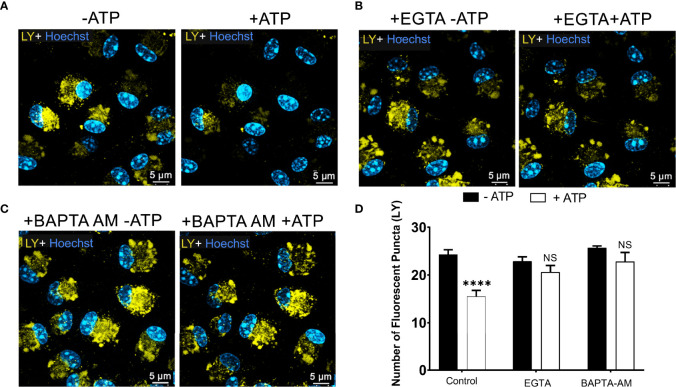
P2X7 Receptor-Induced Lysosomal Rupture depends on Calcium Mobilization. Representative images of peritoneal macrophages labeled with 1 mM of Lucifer Yellow (LY) for 45 min, **(A)** without calcium chelators, **(B)** with 2mM EGTA, and **(C)** 10µM BAPTA-AM chelators, and then treated or not with 5 mM ATP for 10 minutes. **(D)** The number of lysosomes stained with LY was counted before and after treatments. Data are expressed as mean ± SEM of three independent experiments performed in triplicates and analyzed using one-way analysis of variance ****P < 0.0001. Analysis of conditions without ATP vs. after treatment with 5 mM ATP. NS, not significant.

P2X7 receptor activation triggers an increase in intracellular calcium concentration (30). This calcium mobilization induces the opening of the pannexin-1 pore, contributing to the effect of extracellular ATP in endolysosomes and cell permeabilization. Therefore, we tested the effect of calcium signals on ATP-induced endolysosomal leakage. Peritoneal macrophages previously incubated with LY were maintained in an external solution containing 2 mM EGTA, a Ca^2+^ chelator, and observed by confocal microscopy before and during 5 mM ATP treatment for 10 minutes (**Figures 4A, B**). EGTA significantly blocked the effect of extracellular ATP on endolysosome stability (**Figures 4B, D**). The same results were obtained using 10 μM BAPTA-AM, another calcium chelator (**Figures 4C, D**). These findings suggest the relevance of calcium signals for the effect of extracellular ATP on endolysosomal destabilization (*p* < 0.0001).

We have also ascertained that eATP opens Ca^2+^-permeable channels in WT non-stimulated macrophages using fura-2 ratiometric fluorescence imaging (**Supplementary Figures 3A, B**). ATP induced a fast rise in intracellular Ca^2+^ concentration in nearly all cells examined and the Ca^2+^ level decayed very slowly after removal of ATP for several minutes. After changing to a Ca^2+^-free extracellular medium, ATP could not evoke a Ca^2+^ response, demonstrating a requirement of the cation influx. This change was largely reversible since another response could be evoked after washing with the medium containing Ca^2+^, showing the integrity of macrophage cells during the assay (**Supplementary Figures 3A, B**).

The ^10^Panx is described as a pannexin-1 inhibitor that does not interfere directly with ATP-induced P2X7 responses (17). Considering that prolonged rise in intracellular Ca^2+^ induced by ATP could be partly sustained by influx through “large”, non-selective pannexin-1 channels we evaluated the effects of ^10^Panx in ATP-mediated Ca^2+^ responses. The addition of 50 µM ^10^Panx accelerated the decay of the ATP-induced Ca^2+^ response (**Supplementary Figures 4A-C**). Interestingly, in all 15 (of 70) cells which showed a slight initial fast rise followed by a further slow increase in *R* (positive slope), ^10^Panx caused a decay, reversing the trend. This result supports the interpretation that the P2X7-gated Ca^2+^ influx was followed by a secondary Ca^2+^ influx mediated by pannexin-1.

### Possible Involvement of Specific Ion Channels of the Endolysosomal Membrane the P2X7 Receptor- and Pannexin-1-Mediated Lysosomal Leakage

**Figure 5:**
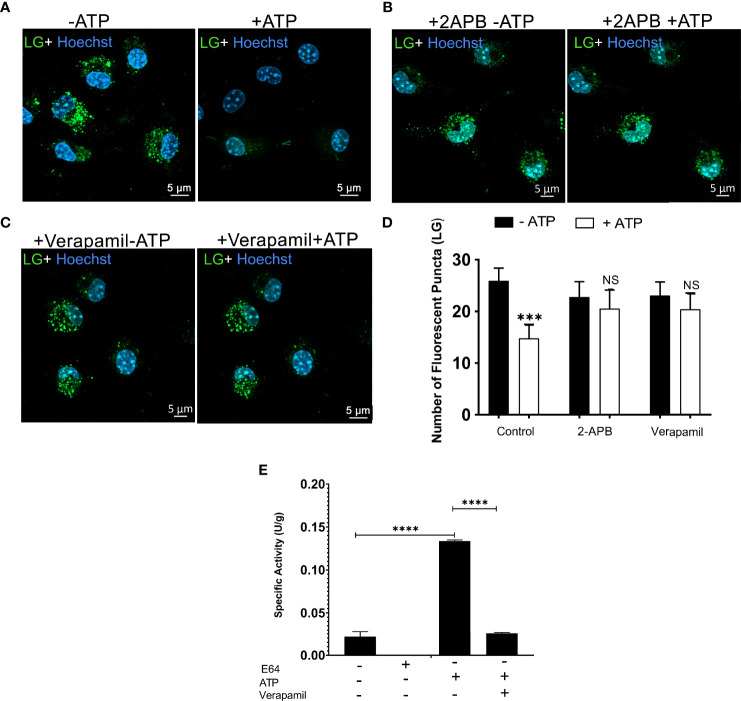
Calcium-Dependent Lysosomal Ion Channels Possible Mediate P2X7 Receptor-induced Lysosomal Rupture. Representative images of WT peritoneal macrophages labeled with 50 nM Lysotracker Green (LY) for 45 min **(A)** without treatments of inhibitors **(B)** WT peritoneal macrophage pre-incubated with 50 µM 2-APB, or **(C)** 50 µM verapamil for 30 minutes, and then treated or not with 5 mM ATP for 10 minutes. **(D)** The number of lysosomes stained with Lysotracker Green was counted before and after treatments. **(E)** Cathepsin activity in supernatants of cells pretreated with 50 µM of verapamil and treated or not with 5 mM ATP for 10 minutes. Data are expressed as mean ± SEM of three independent experiments performed in triplicates and analyzed using one-way analysis of variance ***P < 0.001 and ****P < 0.0001. Analysis of conditions without ATP vs. after treatment with 5 mM ATP **(D, E)**. Analysis of conditions without ATP vs. after treatment with 5 mM ATP and treatment with 5 mM ATP vs. verapamil **(E)**. NS, not significant.

We also hypothesized that calcium-dependent/permeable channels present in the endolysosome membrane, including TRPM2 and TPC channels, would also contribute to eATP-induced organelle leakage. We used the TRPM2 inhibitor (50 μM 2-APB) to test this hypothesis. The 2-APB inhibited ATP-induced lysosome destabilization (**Figures 5A, B**). We also used a TPC channel inhibitor (verapamil 50 μM), and the same result was observed (**Figures 5C, D**). Finally, we determined whether the release of cathepsin induced by ATP treatment would be inhibited by 50 μM verapamil. The TPC inhibitor significantly reduced cathepsin release caused by 5 mM ATP treatment (**Figure 5E**, *p* < 0.0001). These findings suggest that these two calcium-permeable channels might be involved in the mechanism associated with lysosomal leakage triggered by extracellular ATP acting *via* the P2X7 receptor. However, we do not exclude the possibility that P2X7 receptor activation also induces lysosomal alkalinization or exocytosis in these settings, as previously suggested (31, 32).

## Discussion

LMP contributes to the release of cathepsins into the cytoplasm and the production of cytokines *via* activation of the NLRP3 inflammasome activation in macrophages (33, 34). This phenomenon activates various cellular mechanisms resulting in apoptotic, pyroptotic, and necrotic cell death (34). LMP can be triggered by pro-inflammatory agents, including lysosomotropic and damage-associated molecular patterns (2). Evidence suggests that P2X7 receptor activation can cause lysosomal dysfunction, possibly associated with the release of cathepsins and severe inflammatory diseases (19, 20). Nevertheless, the cellular mechanisms by which P2X7 receptor activation induce LMP or leakage are unclear.

The P2X7 receptor is a critical member of the purinergic receptor family concerning immunity (9). This receptor is an essential modulator of inflammatory, infectious, neurodegenerative, and autoimmune diseases (9, 11). P2X7 is a less-sensitive P2 receptor that requires hundreds of micromolar (> 100 µM) to millimolar of extracellular ATP concentrations to become activated (35, 36). Interestingly, we found that the number of fluorescence-labeled endolysosomes and lysosomes diminished only after treating mouse peritoneal macrophages with hundreds of micromolar or millimolar ATP concentrations. This effect was not observed when these cells were treated with low ATP concentrations (50 µM) that do not activate the P2X7 receptor. These results were confirmed by performing the endocytosis assay with YO-PRO^®^-1, a DNA-staining dye. Cells were exposed to YO-PRO^®^-1 and then washed three times before 5 mM ATP treatment. After ATP stimulation YO-PRO^®^-1 was probably released from endocytic vesicles staining the cell nucleus. These findings strongly suggest that the P2X7 receptor is involved in LMP and leakage, as suggested previously (19, 20).

Lopez-Castejon et al. (2010) (20) demonstrated that P2X7 receptor activation in bone marrow-derived macrophages promoted cathepsin B release; this protease was sufficient to degrade the collagen present in the extracellular matrix, promoting inflammation. Hoegen et al. (2011) (37) showed that a pneumococcal pore-forming toxin-induced ATP release in differentiated human THP-1 cells, causing lysosomal destabilization and cathepsin B activation, leading to NLRP3 inflammasome activation and IL-1β production. Similarly, we found that pharmacological inhibition or genetic deletion of the P2X7 receptor blocked extracellular ATP-induced LMP and cathepsin release. Moreover, macrophages from P2X7^-/-^ mice did not show a decrease in fluorescent lysosomes after treatment with 5 mM ATP, and this effect was not seen in WT macrophages pretreated with a P2X7 receptor-specific antagonist A740003. In addition, no increases in cathepsin B activity were observed in the supernatants of P2X7 receptor-deficient or A740003-pretreated macrophages stimulated with ATP, reinforcing the notion that P2X7 receptor induces cathepsin B release. To confirm and visualize the presence of active cathepsin B in the cytoplasm after ATP treatment, we performed endocytosis assays using a fluorogenic substrate for cathepsin B (MR-RR2) with Lysotracker Green (28). After treatment with 5 mM ATP, active cathepsin B left the lysosome and reached the cytoplasm. This can be seen by the increase in the diffuse red staining in the cytoplasm and the decrease in the yellow dots that indicate cresyl violet and lysotracker green colocalization. This result suggests that extracellular ATP could induce cathepsin B escape from lysosomes to the cytoplasm and then to the extracellular medium *via* pannexin-1 hemichannels. However, in this same assay, it was also possible to detect changes in the position of lysosomes from the perinuclear region to the peripheral area of the cell. Although we have data showing endolysosomal content release to cytoplasm using lucifer yellow (data not shown) and the cell cytoplasm diffusely marked in red in the cathepsin B fluorogenic substrate assay (**Figure 2**), we do not rule out the possibility that lysosomal alkalinization is also occurring due to the location of this organelle in the peripheral region, which indicates an increase in luminal pH (31, 32, 38).

Marina-García et al. (2008) (39) proposed that the P2X7 receptor was essential for releasing muramyl dipeptide from endolysosomes into the cytoplasm and the subsequent opening of pannexin-1 channels. These events mediated activation of the NLRP3 inflammasome and induced the release of cytokines in bone marrow-derived macrophages (39, 40). We also observed that peritoneal macrophages incubated with pannexin-1 blockers did not show a significant decrease in the number of fluorescent lysosomes. These blockers also inhibited the release of cathepsin B. These findings suggest that the opening of pannexin-1 channels is essential for ATP-P2X7-induced LMP. As a transmembrane protein, pannexin-1 may act at the membrane of endolysosomes, allowing these organelles’ permeabilization or these pannexin-1 channels to potentiate the Ca^+2^ influx promotes the activation of specific lysosomal calcium channels.

Calcium signaling in the lysosome membrane is essential for the proper functioning of the organelle and ensures the proper functioning of some enzymes and proteins present in the lumen of lysosomes (37). The lysosomal membrane contains ion channels and transporters that maintain the proper gradient and concentration of potassium, sodium, hydrogen, chlorine, and calcium. Some ion channels expressed in the membrane of endolysosomes participate in the regulation of the Ca^+2^ in these compartments, including the TRP family channels (TRPMLs, TRPM2, TRPA1) and the TPC family channels (TPC1, 2) (4, 41)

TRPM2 channel partially contributes to LMP-induced Ca^2+^ influx, K^+^ efflux, and NLRP3 signaling in dendritic cells (33). Furthermore, TRPM2 channel knockout macrophages showed reduced NLRP3 inflammasome activation (approximately 50%) (39, 42). The TPCs are calcium-, sodium-, and hydrogen-permeable ion channels, and their activation can also modulate the activity of the cathepsins in the lumen of lysosomes and endolysosomes (4, 43–45). We found that calcium chelators and blockers of TRPM2 channels and TPC channels inhibited the ATP-P2X7 receptor-induced endolysosomal/lysosomal destabilization and cathepsin B release, suggesting the possible involvement of these channels in this phenomenon in our settings. Although further studies using TRPM2 or TPC knockdown cells are required to confirm these results, the description of these ATP-mediated LMP pathways is relevant for several inflammatory and infectious diseases because ATP is considered a danger signal released from stressed, injured, and activated cells, promoting inflammation (46).

**Figure 6:**
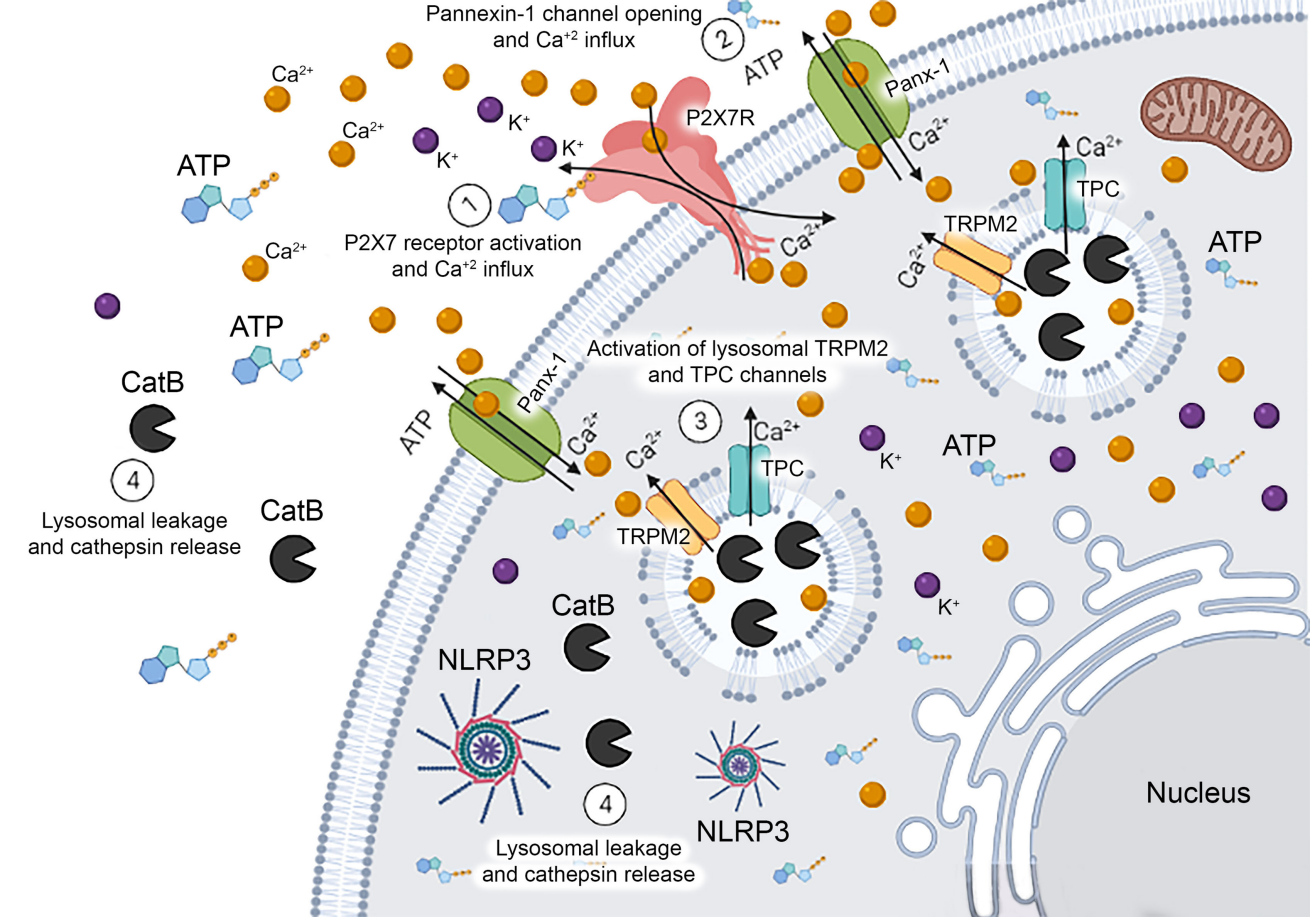
Schematic Representation of Mechanisms Related to Endolysosomal Leakage Induced by P2X7 Receptor Activation. Extracellular ATP activates the P2X7 receptor triggering the opening of the pannexin-1 pore and causing calcium influx. The increase in intracellular calcium concentration might activate TRPM2 and TPC endolysosomal calcium channels inducing the leakage of this organelle and cathepsin B release into the cytoplasm. This protease can activate NLRP3 inflammasome contributing to the release of pro-inflammatory cytokines in macrophages.

In summary, we demonstrated cellular mechanisms by which P2X7 activation induces LMP/leakage and cathepsin B release into the cytoplasm, at least in part (**Figure 6**). These findings have robust relevance because the release of cathepsins is involved in pathophysiological events in various diseases. In addition to the relevance of cathepsin B for the activation of NLRP3 inflammasome and cell death mechanisms, the process of lysosomal leakage induces the release of PAMPs into the cytoplasm or contributes to the escape of antigens and pathogens (47). Interestingly, cathepsin B knockout mice showed severe restriction in the MHC-II-dependent antigen presentation through the cleavage of the class II-associated invariant chain peptide of the invariant chain (Ii) (48–50). In this context, the modulation of LMP and cathepsin B release *via* ATP-P2X7 receptor signaling might interfere in critical inflammatory pathways and antigen presentation mechanisms in immune-related diseases. These findings suggest new molecular targets to modulate lysosomal dysfunction, alkalinization, and permeabilization and open new avenues for drug repurposing and discovery to treat inflammatory, autoimmune, and infectious diseases.

## Data Availability Statement

The raw data supporting the conclusions of this article will be made available by the authors, without undue reservation.

## Ethics Statement

The Commission for the Ethical Use of Research Animals from the Federal University of Rio de Janeiro (UFRJ) approved all experiments (protocol number: IBCCF166/18).

## Author Contributions

SS made substantial contributions to data acquisition, analysis, and interpretation and wrote the manuscript. PP (*in memoriam*) contributed to the concept and design. BH-S, FG, JS, MS, NC, RC-S, and VB-S participated in data acquisition or data analysis and interpretation. EK and LS substantially contributed to the concept, design, data interpretation, and manuscript preparation. All authors critically reviewed the article content and gave final approval of the version to be submitted.

## Conflict of Interest

The authors declare that the research was conducted in the absence of any commercial or financial relationships that could be construed as a potential conflict of interest.

## Publisher’s Note

All claims expressed in this article are solely those of the authors and do not necessarily represent those of their affiliated organizations, or those of the publisher, the editors and the reviewers. Any product that may be evaluated in this article, or claim that may be made by its manufacturer, is not guaranteed or endorsed by the publisher.

## References

[1] Huotari J Helenius A. Endosome Maturation. EMBO J (2011) 30:3481–500. doi: 10.1038/emboj.2011.286 PMC3181477 21878991

[2] Gordon S Martinez-Pomares L. Physiological Roles of Macrophages. Pflugers Arch Eur J Physiol (2017) 469:365–74. doi: 10.1007/s00424-017-1945-7 PMC5362657 28185068

[3] Xu H. Lysosomal Physiology. Annu Rev Physiol (2015) 77:57–80. doi: 10.1146/annurev-physiol-021014-071649 25668017 PMC4524569

[4] Lange I Yamamoto S Partida-Sanchez S Mori Y Fleig A Penner R. TRPM2 Functions as a Lysosomal Ca2+-Release Channel in Cells. Sci Signal (2009) 2:ra23–3. doi: 10.1126/scisignal.2000278 PMC2779714 19454650

[5] Morgan AJ Platt FM Lloyd-Evans E Galione A. Molecular Mechanisms of Endolysosomal Ca 2+ Signalling in Health and Disease. Biochem J (2011) 439:349–74. doi: 10.1042/BJ20110949 21992097

[6] Li P Gu M Xu H. Lysosomal Ion Channels as Decoders of Cellular Signals. Trends Biochem Sci (2019) 44:110–24. doi: 10.1016/j.tibs.2018.10.006 PMC6340733 30424907

[7] Boya P. Lysosomal Function and Dysfunction: Mechanism and Disease. Antioxid Redox Signal (2012) 17:766–74. doi: 10.1089/ars.2011.4405 22098160

[8] Wang F Gómez-Sintes R Boya P. Lysosomal Membrane Permeabilization and Cell Death. Traffic (2018) 19:918–31. doi: 10.1111/tra.12613 30125440

[9] Di Virgilio F Adinolfi E. Extracellular Purines, Purinergic Receptors and Tumor Growth. Oncogene (2017) 36:293–303. doi: 10.1038/onc.2016.206 27321181 PMC5269532

[10] Savio LEB Mello P de A da Silva CG Coutinho-Silva R. The P2X7 Receptor in Inflammatory Diseases: Angel or Demon? Front Pharmacol (2018) 9:52. doi: 10.3389/fphar.2018.00052 29467654 PMC5808178

[11] Coutinho-Silva R Savio LEB. Purinergic Signalling in Host Innate Immune Defence Against Intracellular Pathogens. Biochem Pharmacol (2021) 187:114405. doi: 10.1016/j.bcp.2021.114405 33406411

[12] Coutinho-Silva R Persechini PM. P2Z Purinoceptor-Associated Pores Induced by Extracellular ATP in Macrophages and J774 Cells. Am J Physiol (1997) 273:C1793–800. doi: 10.1152/ajpcell.1997.273.6.C1793 9435482

[13] Ferrari D Pizzirani C Adinolfi E Lemoli RM Curti A Idzko M. The P2X7 Receptor: A Key Player in IL-1 Processing and Release. J Immunol (2006) 176:3877–83. doi: 10.4049/jimmunol.176.7.3877 16547218

[14] Di A Xiong S Ye Z Malireddi RKS Kometani S Zhong M. The TWIK2 Potassium Efflux Channel in Macrophages Mediates NLRP3 Inflammasome-Induced Inflammation. Immunity (2019) 49:56–65. doi: 10.1016/j.immuni.2018.04.032 PMC6051907 29958799

[15] Zumerle S Calì B Munari F Angioni R Di Virgilio F Molon B. Intercellular Calcium Signaling Induced by ATP Potentiates Macrophage Phagocytosis. Cell Rep (2019) 27:1–10. doi: 10.1016/j.celrep.2019.03.011 30943393 PMC6449513

[16] Zhou KQ Green CR Bennet L Gunn AJ Davidson JO. The Role of Connexin and Pannexin Channels in Perinatal Brain Injury and Inflammation. Front Physiol (2019) 27:141. doi: 10.3389/fphys.2019.00141 PMC6400979 30873043

[17] Pelegrin P Surprenant A. Pannexin-1 Mediates Large Pore Formation and Interleukin-1β Release by the ATP-Gated P2X7 Receptor. EMBO J (2006) 25:5071–82. doi: 10.1038/sj.emboj.7601378 PMC1630421 17036048

[18] Dahl G. The Pannexin1 Membrane Channel: Distinct Conformations and Functions. FEBS Lett (2018) 592:3201–9. doi: 10.1002/1873-3468.13115 29802622

[19] Takenouchi T Nakai M Iwamaru Y Sugama S Tsukimoto M Fujita M. The Activation of P2X7 Receptor Impairs Lysosomal Functions and Stimulates the Release of Autophagolysosomes in Microglial Cells. J Immunol (2009) 182:2051–62. doi: 10.4049/jimmunol.0802577 19201858

[20] Lopez-Castejon G Theaker J Pelegrin P Clifton AD Braddock M Surprenant A. P2X 7 Receptor-Mediated Release of Cathepsins From Macrophages Is a Cytokine-Independent Mechanism Potentially Involved in Joint Diseases. J Immunol (2010) 185:2611–9. doi: 10.4049/jimmunol.1000436 20639492

[21] Sekar P Huang DY Hsieh SL Chang SF Lin WW. AMPK-Dependent and Independent Actions of P2X7 in Regulation of Mitochondrial and Lysosomal Functions in Microglia. Cell Commun Signal (2018) 16:1–15. doi: 10.1186/s12964-018-0293-3 30458799 PMC6245559

[22] Campagno KE Mitchell CH. The P2X7 Receptor in Microglial Cells Modulates the Endolysosomal Axis, Autophagy, and Phagocytosis. Front Cell Neurosci (2021) 15:645244. doi: 10.3389/fncel.2021.645244 33790743 PMC8005553

[23] Bayati A Kumar R Francis V McPherson PS. SARS-CoV-2 Infects Cells After Viral Entry *via* Clathrin-Mediated Endocytosis. J Biol Chem (2021) 296:1–12. doi: 10.1016/j.jbc.2021.100306 PMC7816624 33476648

[24] Fairbairn IP Stober CB Kumararatne DS Lammas DA. ATP-Mediated Killing of Intracellular Mycobacteria by Macrophages Is a P2X(7)-Dependent Process Inducing Bacterial Death by Phagosome-Lysosome Fusion. J Immunol (2001) 167:3300–7. doi: 10.4049/jimmunol.167.6.3300 11544318

[25] Solle M Labasi J Perregaux DG Stam E Petrushova N Koller BH. Altered Cytokine Production in Mice Lacking P2X(7) Receptors. J Biol Chem (2001) 276:125–32. doi: 10.1074/jbc.M006781200 11016935

[26] Swanson JA Yirinec BD Silverstein SC. Phorbol Esters and Horseradish Peroxidase Stimulate Pinocytosis and Redirect the Flow of Pinocytosed Fluid in Macrophages. J.Cell Biol (1985) 100:851–9. doi: 10.1083/jcb.100.3.851 PMC2113515 3972898

[27] Page E Goings GE Upshaw-Earley J Hanck DA. Endocytosis and Uptake of Lucifer Yellow by Cultured Atrial Myocytes and Isolated Intact Atria From Adult Rats Regulation and Subcellular Localization. Circ Res (1994) 75:335–46. doi: 10.1161/01.res.75.2.335 8033344

[28] Boonacker E Van Noorden CJF. Enzyme Cytochemical Techniques for Metabolic Mapping in Living Cells, With Special Reference to Proteolysis. J Histochem Cytochem (2001) 49:1473–86. doi: 10.1177/002215540104901201 11724895

[29] Idziorek T Estaquier J De Bels F Ameisen JC. YOPRO-1 Permits Cytofluorometric Analysis of Programmed Cell Death (Apoptosis) Without Interfering With Cell Viability. J Immunol Methods (1995) 185:249–58. doi: 10.1016/0022-1759(95)00172-7 7561136

[30] Murgia M Hanau S Pizzo P Rippa M Di Virgilio F. Oxidized ATP. An Irreversible Inhibitor of the Macrophage Purinergic P2Z Receptor. J Biol Chem (1993) 268:8199–203. doi: 10.1016/S0021-9258(18)53082-9 8463330

[31] Guha S Baltazar GC Coffey EE Tu LA Lim JC Beckel JM. Lysosomal Alkalinization, Lipid Oxidation, and Reduced Phagosome Clearance Triggered by Activation of the P2X7 Receptor. FASEB J (2013) 27(11):4500–9. doi: 10.1096/fj.13-236166 PMC3804754 23964074

[32] Gutiérrez-Martín Y Bustillo D Gómez-Villafuertes R Sánchez-Nogueiro J Torregrosa-Hetland C Binz T. P2X7 Receptors Trigger ATP Exocytosis and Modify Secretory Vesicle Dynamics in Neuroblastoma Cells. J Biol Chem (2011) 286(13):11370–81. doi: 10.1074/jbc.M110.139410 PMC3064193 21292765

[33] Katsnelson MA Lozada-Soto KM Russo HM Miller BA Dubyak GR. NLRP3 Inflammasome Signaling Is Activated by Low-Level Lysosome Disruption But Inhibited by Extensive Lysosome Disruption: Roles for K+ Efflux and Ca2+ Influx. Am J Physiol - Cell Physiol (2016) 311:C83–C100. doi: 10.1152/ajpcell.00298.2015 27170638 PMC4967136

[34] Yu X Quan J Long W Chen H Wang R Guo J. LL-37 Inhibits LPS-Induced Inflammation and Stimulates the Osteogenic Differentiation of BMSCs *via* P2X7 Receptor and MAPK Signaling Pathway. Exp Cell Res (2018) 372:178–87. doi: 10.1016/j.yexcr.2018.09.024 30287143

[35] Surprenant A Rassendren F Kawashima E North RA Buell G. The Cytolytic P2Z Receptor for Extracellular ATP Identified as a P2X Receptor (P2X7). Sci (80-.) (1996) 272:735–8. doi: 10.1126/science.272.5262.735 8614837

[36] Ferrari D Chiozzi P Falzoni S Dal Susino M Melchiorri L Baricordi OR. Extracellular ATP Triggers IL-1 Beta Release by Activating the Purinergic P2Z Receptor of Human Macrophages. J Immunol (1997) 159:1451–8. 9233643

[37] Hoegen T Tremel N Klein M Angele B Wagner H Kirschning C. The NLRP3 Inflammasome Contributes to Brain Injury in Pneumococcal Meningitis and Is Activated Through ATP-Dependent Lysosomal Cathepsin B Release. J Immunol (2011) 187:5440–51. doi: 10.4049/jimmunol.1100790 22003197

[38] Johnson DE Ostrowski P Jaumouillé V Grinstein S. The Position of Lysosomes Within the Cell Determines Their Luminal pH. J Cell Biol (2016) 212(6):677–92. doi: 10.1083/jcb.201507112 PMC4792074 26975849

[39] Marina-García N Franchi L Kim Y-G Miller D McDonald C Boons G-J. Pannexin-1-Mediated Intracellular Delivery of Muramyl Dipeptide Induces Caspase-1 Activation *via* Cryopyrin/NLRP3 Independently Nod2. J Immunol (2008) 180:4050–7. doi: 10.4049/jimmunol.180.6.4050 18322214

[40] Franchi L Eigenbrod T Muñoz-Planillo R. The Inflammasome: A Caspase-1-Activation Platform That Regulates Immune Responses and Disease Pathogenesis. Nat Immunol (2009) 10:241–7. doi: 10.1038/ni.1703 PMC2820724 19221555

[41] Cao F Hu LQ Yao SR Hu Y Wang DG Fan YG. P2X7 Receptor: A Potential Therapeutic Target for Autoimmune Diseases. Autoimmun Rev (2019) 18:767–77. doi: 10.1016/j.autrev.2019.06.009 31181327

[42] Zhong Z Zhai Y Liang S Mori Y Han R Sutterwala FS. TRPM2 Links Oxidative Stress to NLRP3 Inflammasome Activation. Nat Commun (2013) 4:1611–35. doi: 10.1038/ncomms2608 PMC3605705 23511475

[43] Shen J. Function and Dysfunction of Presenilin. Neurodegener Dis (2014) 13:61–3. doi: 10.1159/000354971 PMC4000081 24107444

[44] Grimm C Butz E Chen CC Wahl-Schott C. And Biel, M. (2017). From Mucolipidosis Type IV to Ebola: TRPML and Two-Pore Channels at the Crossroads of Endolysosomal Trafficking and Disease. Cell Calcium (2017) 67:148-55. doi: 10.1016/j.ceca.2017.04.003 28457591

[45] Patel S Kilpatrick BS. Two-Pore Channels and Disease. Biochim. Biophys Acta - Mol Cell Res (2018) 1865:1678–86. doi: 10.1016/j.bbamcr.2018.05.004 PMC6162333 29746898

[46] Lawrence RE Zoncu R. The Lysosome as a Cellular Centre for Signalling, Metabolism and Quality Control. Nat Cell Biol (2019) 21:133–42. doi: 10.1038/s41556-018-0244-7 30602725

[47] Oyarzún JE Lagos J Vázquez MC Valls C de la Fuente C Yuseff MI. Lysosome Motility and Distribution: Relevance in Health and Disease. Biochim Biophys Acta - Mol Basis Dis (2019) 1865:1076–87. doi: 10.1016/j.bbadis.2019.03.009 30904612

[48] Matsunaga Y Saibara T Kido H Katunuma N. Participation of Cathepsin B in Processing of Antigen Presentation to MHC Class II. FEBS Lett (1993) 324:325–30. doi: 10.1016/0014-5793(93)80144-J 8405375

[49] Honey K Rudensky AY. Lysosomal Cysteine Proteases Regulate Antigen Presentation. Nat Rev Immunol (2003) 3:472–82. doi: 10.1038/nri1110 12776207

[50] Campden RI Zhang Y. The Role of Lysosomal Cysteine Cathepsins in NLRP3 Inflammasome Activation. Arch Biochem Biophys (2019) 670:32–42. doi: 10.1016/j.abb.2019.02.015 30807742

