# Supplementary material for: P2X7 Receptor Triggers Lysosomal Leakage Through Calcium Mobilization in a Mechanism Dependent on Pannexin-1 Hemichannels
Source: Front Immunol. 2022 Feb 9;13:752105. doi: 10.3389/fimmu.2022.752105 (PMC8863609; doi:10.3389/fimmu.2022.752105)
Supplement: Supplementary file 1 [file DataSheet_1.docx]

Supplementary Material

***Santos SACS, Persechini PM, Henriques-Santos BM, Bello-Santos VG, Castro NG, Costa de Sousa J, Genta FA, Santiago M, Coutinho-Silva R, Savio LEB and Kurtenbach E (2022) P2X7 Receptor Triggers Lysosomal Leakage Through Calcium Mobilization in a Mechanism Dependent on Pannexin-1 Hemichannels. Front. Immunol. 13:752105. doi: 10.3389/fimmu.2022.752105***

**
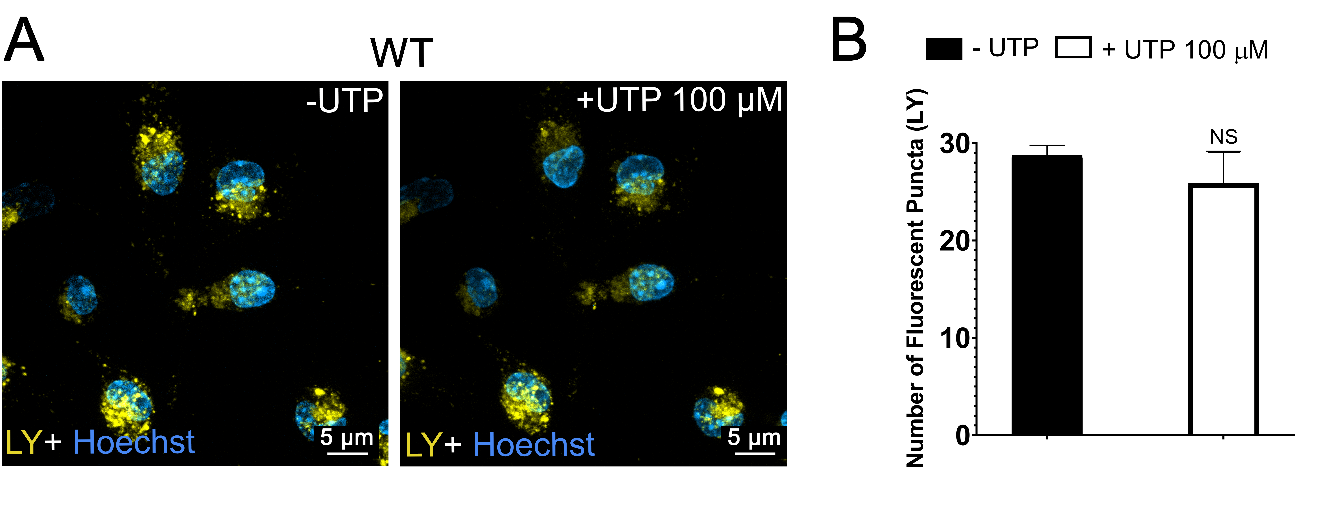
**

**Supplementary Figure 1. Extracellular UTP Is Not Able to Induce Endolysosomal Leakage.** Representative images of peritoneal macrophages from C57Bl/6 WT labeled with (A) 1 mM of Lucifer Yellow (LY) for 45 min and then treated with 100 µM UTP at 37 °C for 10 min. (B) The number of fluorescent puncta per cell stained with Lucifer Yellow was counted before and after UTP treatment. Scale bar, 5 µM; original magnification 100x. Data are expressed as mean ± SEM of three independent experiments performed in triplicate. (B) Analysis with the t-test to compare the condition of 10 min after treatment with UTP vs. without treatment with UTP. NS = not significant.


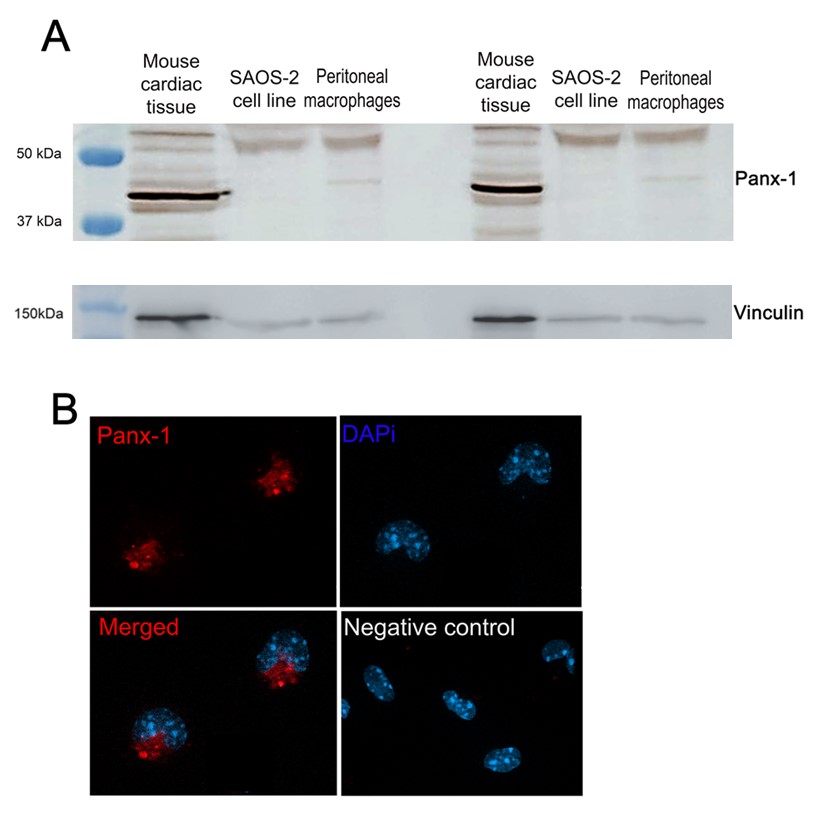


**Supplementary Figure 2: Pannexin-1 Expression In Non-stimulated Peritoneal Macrophages.** (A)Western Blot images of pannexin-1(~48 kDa) and vinculin (~116 kDa) (loading control) expression in mouse heart tissue (positive control), SAOS-2 cells (negative control), and peritoneal macrophages. (B) Immunocytochemistry of representative images of peritoneal macrophages from WT C57Bl/6 incubated (overnight at 4°C) with the primary antibodies anti-pannexin-1. Scale bar, 5 µM; original magnification 100x.

**Supplementary Figure 3.** **ATP Induces Ca^2+^ Influx In WT Mice Peritoneal Macrophages.** Fura-2 fluorescence ratio (*R*) traces from single cells (grey), and their average (red) from a representative WT macrophage culture. (A) show a fast rise in intracellular Ca^2+^ levels when exposed to 5 mM ATP (black horizontal bars) in physiological extracellular solution containing 1 mM CaCl_2_ (control solution). The traces were baseline-subtracted to facilitate visualization of the first response. Removal of extracellular Ca^2+^ (0-Ca^2+^ physiological extracellular solution containing 2 mM EGTA, lower black horizontal bar) eliminated the response to a 2^nd^ pulse of 5 mM ATP. After changing back to control solution, a 3^rd^ pulse of 5 mM ATP evoked a response in all cells. (B) All pairwise differences were significant (P < 0.0001, Kruskal-Wallis ANOVA and Mann-Whitney tests).

**Supplementary Figure 4.** **Pannexin-1 Contributes to the ATP-induced Rise in Intracellular Ca^2+^.** Fura-2 fluorescence ratio (*R*) traces from single cells (grey) and their average (red) from a representative WT macrophage culture (A) show sustained intracellular Ca^2+^ levels during prolonged exposure to 5mM ATP in physiological extracellular solution. The addition of ^10^Panx (50 µM final, with 5 mM ATP) reduced the Ca^2+^ levels. The rate of change of the Ca^2+^ signal (B, C) was estimated by linear regression in two 200-s data segments, one just after the addition of ATP and another 2 min after the addition of ^10^Panx (red brackets above traces in A); the change in rate was significant (P < 0.0001, Wilcoxon test). The cells where the Ca^2+^ signal initially rose showed clear inversion of the trend after adding ^10^Panx (red symbols in C).
